# Effects of Annealing on GaAs/GaAsSbN/GaAs Core-Multi-shell Nanowires

**DOI:** 10.1186/s11671-016-1265-4

**Published:** 2016-02-01

**Authors:** Pavan Kasanaboina, Manish Sharma, Prithviraj Deshmukh, C. Lewis Reynolds, Yang Liu, Shanthi Iyer

**Affiliations:** Department of Electrical and Computer Engineering, North Carolina A&T State University, Greensboro, NC 27411 USA; Nanoengineering, Joint School of Nanoscience and Nanoengineering, NC A&T State University, Greensboro, NC 27401 USA; Department of Materials Science and Engineering, North Carolina State University, Raleigh, NC 27695 USA

**Keywords:** Dilute nitrides, Nanowires, Annealing effects, Schottky barrier, Point defects

## Abstract

The effects of *ex-situ* annealing in a N_2_ ambient on the properties of GaAs/GaAsSbN/GaAs core-multi-shell nanowires on Si (111) substrate grown by self-catalyzed molecular beam epitaxy (MBE) are reported. As-grown nanowires exhibit band edge emission at ~0.99 eV with a shoulder peak at ~0.85 eV, identified to arise from band tail states. A large red shift of 7 cm^−1^ and broadened Raman spectra of as-grown nanowires compared to that of non-nitride nanowires confirmed phonon localization at N-induced localized defects. On annealing nanowires to 750 °C, there was no change in the planar defects in the nanowire with respect to the as-grown nanowire; however, vanishing of the photoluminescence (PL) peak corresponding to band tail states along with enhanced band edge PL intensity, recovery of the Raman shift and increase in the Schottky barrier height from 0.1 to 0.4 eV clearly point to the efficient annihilation of point defects in these GaAsSbN nanowires. A significant reduction in the temperature-induced energy shift in the annealed nanowires is attributed to annihilation of band tail states and weak temperature dependence of N-related localized states. The observation of room temperature PL signal in the 1.3 μm region shows that the strategy of adding small amounts of N to GaAsSb is a promising route to realization of efficient nanoscale light emitters with reduced temperature sensitivity in the telecommunication wavelength region.

## Background

Dilute nitride III–V semiconductor alloys in thin films with variable bandgap have been on the extensively studied material systems [1–10] for optical telecommunications applications. Introduction of very low nitrogen concentrations is sufficient to modulate the band gap of such systems over a wide range. Amongst the possible III–V-based dilute nitride materials, GaAsSbN systems in the thin film configuration have been proven to be one of the promising systems for the spectral window of 1.3–1.55 μm [1]. Simultaneous reduction in the band gap with lattice parameter and also independent tuning of conduction and valence band offsets by the N and Sb constituents, respectively, are the distinguishing features of this system. However, large disparity in the atomic radius of N and Sb creates N-induced defects and compositional disorder leading to non-radiative recombination centers, which adversely affect the optoelectronic properties of the thin film. It is well established that considerable improvement in the optical properties occurs on annealing due to the annihilation of N-related centers. Reducing dimensionality further to nanowires, for example, may prove to be advantageous for this system in terms of efficient annihilation of point defects and enhanced photon collection due to the large surface to volume ratio.

However, little work has been reported on dilute nitride GaAsSbN nanowires due to the complexities involved in growth of quaternary alloy nanowires. Recently, we have reported successful growth of self-catalyzed GaAs/GaAsSbN/GaAs core-multi-shell nanowires emitting at ~1.3 μm [11]. These nanowires exhibited planar defects in addition to point defects, which is in contrast to thin films where only the latter dominates [12]. Thus, it is necessary to investigate the effect of defects and annealing on dilute nitride nanowires to understand and improve the optical properties.

In this work, we present the effects of *ex-situ* rapid thermal annealing (RTA) on the optical properties of GaAs/GaAsSbN/GaAs nanowires grown by self-catalyzed molecular beam epitaxy (MBE). Various characterization techniques, namely, transmission electron microscopy (TEM), low temperature μ-photoluminescence (PL), Raman spectroscopy and current (I)-voltage (V) measurements were used to ascertain the nature of defects being annihilated. Temperature dependent PL was examined to understand the effects of localized states and recombination mechanisms.

## Methods

### Nanowire Growth and Characteristic Analyses

The growth of dilute nitride GaAs/GaAsSbN/GaAs core-shell nanowires was carried out on Si (111) substrates by plasma-assisted MBE. GaAs core nanowires were grown at 620 °C with Ga as a catalyst and an As flux with a beam equivalent pressure (BEP) of 4.8 × 10^−6^ Torr. The growth temperature was then lowered, and GaAsSbN shell growth was initiated at 540 °C by opening the Sb and N shutters. A constant Sb BEP of 1.4 × 10^−6^ Torr and N BEP of 1.8 × 10^−7^ were used for the quaternary shell. Both the Sb and N shutters were closed for growth of the final GaAs shell at 540 °C. Detailed growth procedures and characterization techniques are provided in our previous reports [11, 13, 14]. Basically, TEM, PL, and Raman were used to reveal differences in these nanowires before and after annealing. All the nanowires were annealed in N_2_ ambient using a Jipelec JetFirst 100 RTA for 30 s. For I–V measurements on an ensemble of nanowires, the array of nanowires were initially spun with polymethylmethacrylate (PMMA) which was plasma etched to 200 nm in order to expose the tips of the nanowires. The top contact was Ti (50 nm)/Au (200 nm) while the back contact was Ti (200 nm). To avoid possible short circuits at the sample edges, the top contacts were deposited using a shadow mask consisting of an array of 1-mm-diameter circular apertures to produce discrete contact pads. Then, a contact on the bottom of the substrate was deposited by electron beam evaporation. Electrical measurements were performed at room temperature using a Keithley 4200 characterization system by a two-probe method.

## Results and Discussion

**Fig. 1:**
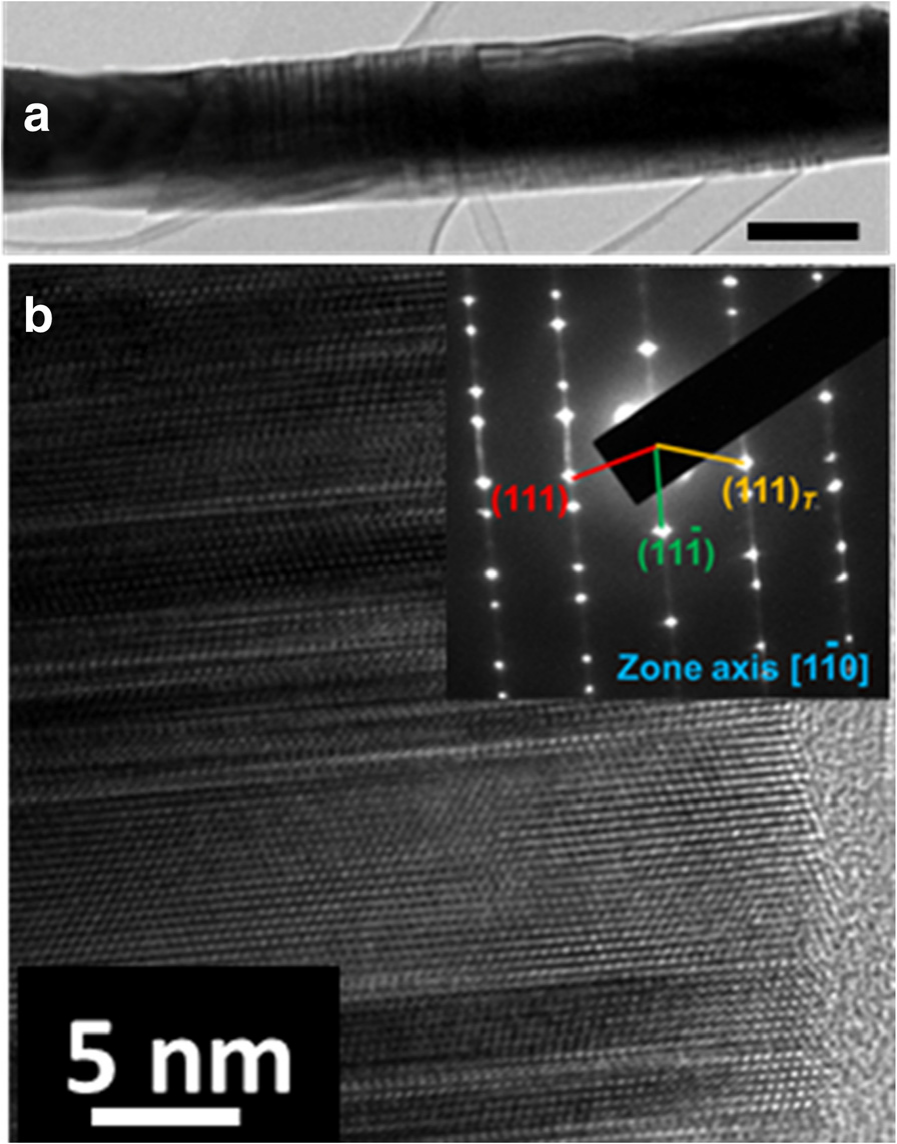
**a** Bright field TEM image of annealed GaAs/GaAsSbN/GaAs core-multi-shell nanowires (*scale bar*: 200 nm). **b** HRTEM and SAED pattern (*inset*) of the nanowire reveal the presence of planar defects and a ZB structure of the nanowires

Figure 1a displays a bright field TEM image of a post growth GaAs/GaAsSbN/GaAs core-shell nanowire annealed at 750 °C. The high-resolution TEM (HR-TEM) image and associated selected area electron diffraction (SAED) pattern confirm the zinc-blende (ZB) structure of the nanowire (Fig. 1b). The existence of twins and stacking faults in these nanowires indicate that annealing does not affect the planar defects present in the as-grown nanowires as described in an earlier publication [11]. This observation is not surprising as it is well known that annealing dilute nitride thin films primarily annihilates N-related point defects. Since it is difficult to assess the presence/absence of point defects in TEM, any inference on their relevance must be based on indirect evidence provided by different techniques, namely, PL, Raman, and I–V measurements as discussed below.

**Fig. 2:**
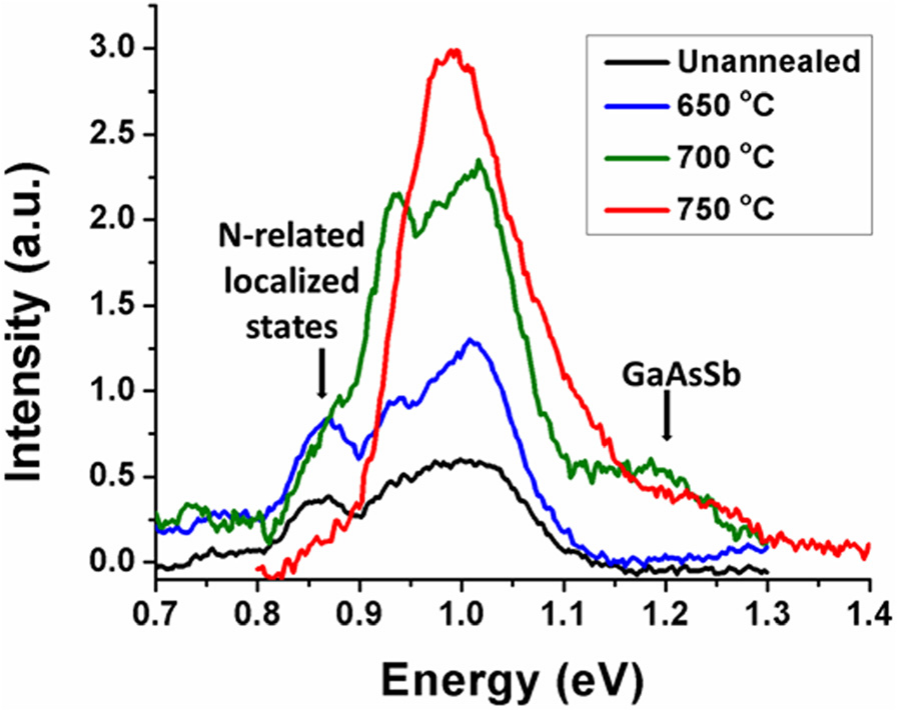
4 K PL spectra of unannealed and annealed dilute nitride GaAs/GaAsSbN/GaAs core-multi-shell nanowires at different temperatures

Figure 2 shows the 4 K PL spectra of GaAs/GaAsSbN/GaAs core-multi-shell nanowires annealed at different temperatures of 650, 700, and 750 °C and compared with unannealed nanowires. The Sb composition in the nanowires is ~10 at.% as estimated from energy dispersive X-ray spectroscopy (EDXS). As-grown unannealed nanowires exhibit two characteristic PL peaks at ~0.99 and ~0.85 eV that are associated with the band to band transition and band tailing, respectively, in dilute nitride III–V systems [8, 15, 16].

Contributors to band tail-induced states include compositional fluctuations, localized defect states, and inhomogeneous lattice deformation [17]. With increasing annealing temperature, the intensity of band to band emission increases by fivefold with a corresponding decrease in the full width at half maxima (FWHM) of the PL peak while the intensity of the N-related defect peak at 0.85 eV gradually diminishes. This latter peak ultimately vanishes for nanowires annealed at 750 °C. These are typical signatures for annihilation of N-related defects, which results from significant reduction in the density of recombination centers responsible for non-radiative processes as in thin films. Suppression of these non-radiative centers facilitates PL emission from higher energy states, which leads to increased PL intensity [8, 9, 16, 18] with increasing annealing temperature. An emission peak at 0.93 eV is also observed in all the samples including unannealed ones. The origin of this peak is not clearly understood; however, it is likely related to compositional fluctuations originating from N incorporation as it merges with the band-band peak for nanowires annealed at 750 °C. In addition, evolution of a distinct PL peak at 1.2 eV at 700 °C which becomes a hump at higher annealing temperature of 750 °C corresponds to a band to band transition of the host non-nitride composition of GaAsSb with 10 at.% of Sb.

**Fig. 3:**
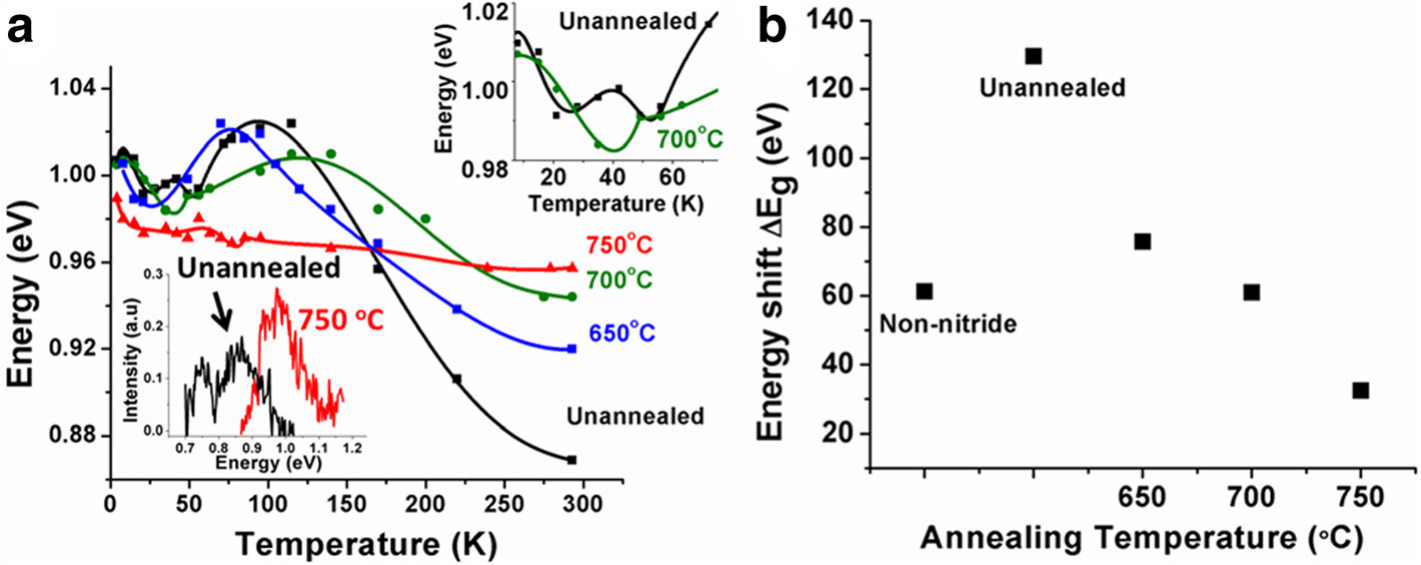
**a** Temperature-dependent PL peak energies (*inset*: room temperature PL spectra of unannealed and 750 °C annealed nanowires). **b** Total energy shift ΔE_g_ (4–300 K) for different annealing temperatures

Temperature dependence of PL emission was studied for all samples, as shown in Fig. 3, to achieve a better understanding of N-induced localized states and recombination mechanisms. All nanowires exhibit the characteristic red-blue-red shift in PL peak energy with temperature, which is known as “S-curve” behavior that is commonly observed in dilute nitride system [8, 9, 16]. This behavior is attributed to exciton localization in band tail states [19–22] due to potential fluctuations. For T < 100 K, radiative recombination is largely determined by localized excitons with free carrier recombination becoming dominant for T > 100 K. This has been commonly explained in dilute nitride thin films [16, 23] as follows.

In the low-temperature regime, with rising temperature, the excitons confined in the local potential minimum obtain sufficient thermal energy to surmount small barriers and thus relaxing to lower energy states. The recombination of these excitons is responsible for the reduced PL emission energy causing an initial red shift. With further increase in temperature, the excitons gain sufficient energy to populate the higher energy band tail states, and the recombination from these states is responsible for the observed blue shift in the band gap. For temperatures in the region of 75 to 140 K, depending on the sample, corresponding to the highest PL peak energy, the excitons are delocalized. Beyond this temperature, free carrier recombination dominates, and the regular temperature-induced band gap shrinkage occurs due to the electron-phonon interaction and lattice relaxation. Although the overall temperature behavior of the PL in thin films and nanowires is the same, in the nanowires, an additional red-blue shift is observed around 50 K. Such an additional feature is normally reported to be due to splitting of the heavy hole (HH) and light hole (LH) leading to the corresponding excitonic transitions [24]. However, the PL spectra shown in Fig. 2 did not exhibit any distinct peaks that would correspond to LH and HH transitions. X-ray diffraction of the annealed GaAsSbN nanowires (not shown here) exhibited only (111) GaAs peak and did not exhibit any other distinct peak as reported previously [11] on as-grown nitride core-shell nanowires. This can be considered as evidence of a lattice-matched GaAs/GaAsSbN/GaAs core-shell structure, which suggests that any contribution of a strain component to the splitting of HH and LH is negligible. We, therefore, speculate and assign the additional low-temperature feature to the differences in the electron-phonon interaction with the HH and LH excitons becoming more pronounced for lower dimensional structures [25] although the energy splitting between HH and LH excitons may be small.

The pronounced S-curve for unannealed nanowires and nanowires annealed at 650 °C suggests a strong localization energy, which is measured to be 31–42 meV. The localization energy reduces to 18.6 and 8 meV with increasing annealing temperature to 700 and 750 °C, respectively, which is indicative of efficient annihilation of band tail states leading to decreased potential fluctuations. This is also further supported by the increase in the PL intensity and narrowing of the full width at half maxima (FWHM) of the PL spectra (Fig. 2). Another dramatic change that is observed with increasing annealing temperature is a reduction in the energy shift between 4 and 300 K, ΔE_g_ (4–300 K), 129 meV for unannealed nanowires to 32.4 meV for the samples annealed at 750 °C as shown in Fig. 3b. These Δ*E*_*g*_ values are consistent with our prior work on GaAsSbN quantum wells (QWs) where in situ annealing in an As ambient corresponded to efficient annihilation of N-related centers [9, 16]. In unannealed and at the lower annealing temperature of 650 °C, the localized N level is coupled with extended states of the conduction band [26, 27]. This can be described by a band anti-crossing (BAC) model and exhibits the characteristic temperature-induced band gap shrinkage. In the annealed nanowires where the band states are efficiently annihilated, the temperature-independent characteristic of the N level prevails. A low activation energy for point defects has been cited as the reason for the annihilation of point defects caused by the movement of the lattice atoms on annealing below a critical temperature [28]. Our data are consistent with an optimum annealing temperature in the range of 700–750 °C for effective annihilation of point defects in a thin film configuration [8, 29]. These are very promising results and can have favorable impact in the temperature-induced shift in device characteristics. The room temperature PL of these samples is in the 1.3 μm region (Fig. 3a inset), the wavelength of great interest for photonic integrated circuits.

**Fig. 4:**
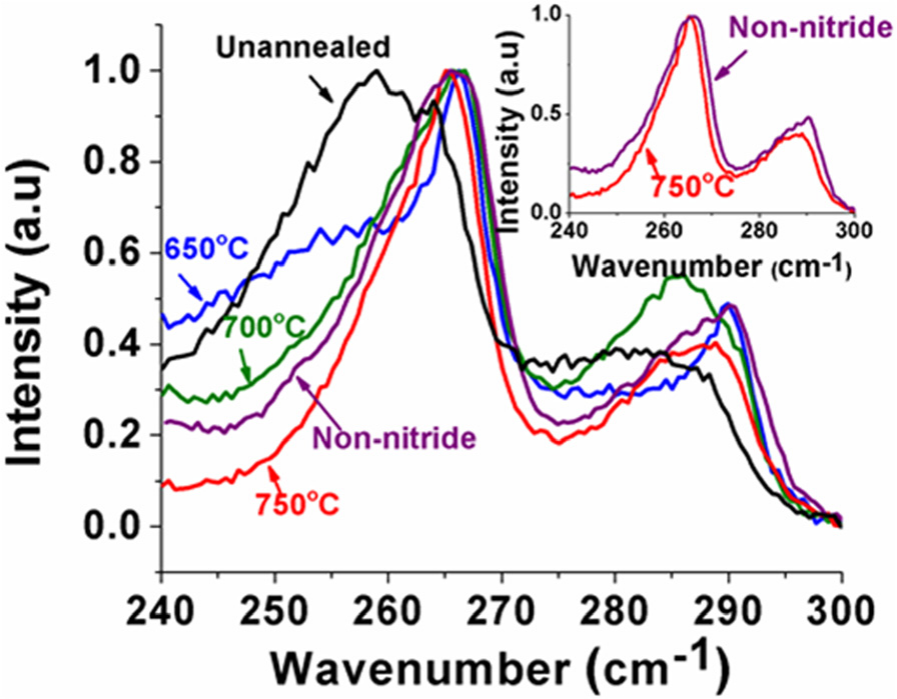
Raman spectra of reference non-nitride, unannealed, and annealed dilute nitride GaAs/GaAsSbN/GaAs core-multi-shell nanowires

Figure 4 displays the Raman spectra of annealed samples and compared to the reference as-grown nanowires. The Raman spectrum of as-grown dilute nitride nanowires is broader and exhibits a large red shift of 7 cm^−1^ compared to that of reference non-nitride nanowires. The origin of this red shift has been discussed in detail earlier papers [11, 30] and has been attributed to phonon localization at point defects. The line shape is also more symmetric for as-grown nanowire. On annealing, the Raman signal reverts back to the reference Raman spectra for non-nitride GaAsSb nanowires and even exhibits the asymmetric line shape. For 750 °C annealed nanowires, the FWHM is also reduced. The change in line shape is representative of the dominance of planar defects over point defects [11]. Thus, Raman data provides additional strong evidence for annihilation of the point defects on annealing.

**Fig. 5:**
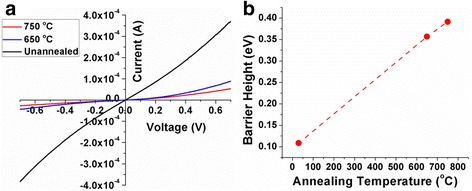
**a** I–V measurements on dilute nitride nanowires annealed at different temperatures. **b** Variation of Schottky barrier height with annealing temperature

Finally, I–V measurements were also conducted to provide more insight into the nature of the defects annihilated. Figure 5a displays I–V measurements on ensembles of nanowires annealed at different temperatures. The I–V characteristic is symmetric around the origin, and for a given voltage, nanowires annealed at 750 °C exhibit the lowest current compared to unannealed and nanowires annealed at 650 °C. Assuming the two contacts at the two ends of the nanowires to be Schottky contacts, the barrier height at these contacts have been determined by best fit to the experimental I–V curve using a Matlab based program applied to a metal-semiconductor-metal (M-S-M) model [31, 32]. As shown in Fig. 5b, the barrier height increases from ~0.1–0.4 eV with increasing annealing temperature from room temperature to 750 °C, respectively. The increase in the barrier height with annealing indicates that charge transfer at the metal-semiconductor interface for as-grown nanowires is due to trap-assisted tunneling. Further, a high concentration of point defects that are associated with shallow donors also promotes enhanced electrical transport [33]. Thus, higher electrical conduction can be viewed as evidence for the presence of a high point defect density in the nanowires. Therefore, the I–V characteristics provide additional support for point defect annihilation in the 750 °C annealed nanowires, which is consistent with the PL and Raman results as discussed earlier. Generally, dilute nitrides are commonly known to exhibit lower Schottky barrier heights due to the effects of interface states, series resistance, tunneling process, and non-uniformity distribution of the interfacial charge [34]. It has been reported that depending on the density of the defects at the metal-semiconductor interface and contact material work function, the Schottky barrier height can vary from 0.36–0.95 eV [35].

## Conclusions

In conclusion, the effect of *ex-situ* annealing of GaAs/GaAsSbN/GaAs core-multi-shell nanowires in a N_2_ ambient has been investigated. With a shoulder at lower energy attributed to band tail states, 4 K μ-PL spectra of the unannealed nanowires display a peak at ~0.99 eV. Raman spectra of as-grown dilute nitride nanowires exhibited a large red shift of 7 cm^−1^ and were broadened in comparison to that of reference non-nitride nanowires, confirming phonon localization at N-induced localized defects. Rapid thermal annealing of these nanowires in a N_2_ ambient has been employed to annihilate various N-induced defects. With increase in annealing temperature from 650–750 °C, the PL peak at lower energy corresponding to band tail states vanishes and also the Raman spectra revert back towards the reference spectra, which are considered as clear evidences of annihilation of N-related defects. Significant increase in the Schottky barrier height of the nanowires annealed at elevated temperature also provided further support to the above inference. The temperature-dependent PL spectra exhibited the well-known S-curve behavior, a characteristic of dilute nitride material systems and a signature of exciton localization. Further, room temperature emission and suppression of the temperature-induced band gap in the annealed nanowires are the added attractive attributes offered by these dilute nitride nanowires towards nanoscale optoelectronic device applications.
